# Development of User-Friendly Wearable Electronic Textiles for Healthcare Applications

**DOI:** 10.3390/s18082410

**Published:** 2018-07-25

**Authors:** Kai Yang, Katie Meadmore, Chris Freeman, Neil Grabham, Ann-Marie Hughes, Yang Wei, Russel Torah, Monika Glanc-Gostkiewicz, Steve Beeby, John Tudor

**Affiliations:** 1 Electronics and Computer Science, University of Southampton, Southampton SO17 1BJ, UK; cf@ecs.soton.ac.uk (C.F.); njg@ecs.soton.ac.uk (N.G.); y.wei@soton.ac.uk (Y.W.); rnt@ecs.soton.ac.uk (R.T.); mgg1y14@soton.ac.uk (M.G.-G.); spb@ecs.soton.ac.uk (S.B.); mjt@ecs.soton.ac.uk (J.T.); 2 Faculty of Health Sciences, University of Southampton, Southampton SO17 1BJ, UK; K.Meadmore@soton.ac.uk (K.M.); A.Hughes@soton.ac.uk (A.-M.H.)

**Keywords:** wearable, e-textiles, printed electronics, electrode, functional electrical stimulation (FES), rehabilitation, muscle stimulation

## Abstract

This paper presents research into a user-friendly electronic sleeve (e-sleeve) with integrated electrodes in an array for wearable healthcare. The electrode array was directly printed onto an everyday clothing fabric using screen printing. The fabric properties and designed structures of the e-sleeve were assessed and refined through interaction with end users. Different electrode array layouts were fabricated to optimize the user experience in terms of comfort, effectivity and ease of use. The e-sleeve uses dry electrodes to facilitate ease of use and the electrode array can survive bending a sufficient number of times to ensure an acceptable usage lifetime. Different cleaning methods (washing and wiping) have been identified to enable reuse of the e-sleeve after contamination during use. The application of the e-sleeve has been demonstrated via muscle stimulation on the upper limb to achieve functional tasks (e.g., hand opening, pointing) for eight stroke survivors.

## 1. Introduction

E-textiles are advanced textiles that include electronic functionality ranging from conductive yarns/tracks [1,2,3,4] to sensing/actuating [5,6,7,8,9], communications [10,11] and signal processing [12,13]. Emerging advanced e-textile technologies offer rich opportunities to push the boundaries of wearable healthcare applications by improving the user experience (e.g., comfort, ease of use, unobtrusiveness) thus motivating the user to adhere to the recommended usage.

Electrodes are fundamental elements used in numerous healthcare devices to measure the body’s bio-potentials, for example, electrocardiography (ECG), electroencephalography (EEG) and electromyography (EMG). They are also being used in therapeutic healthcare devices such as transcutaneous electrical nerve stimulation (TENS) for pain relief and functional electrical stimulation (FES) for muscle exercise and rehabilitation. Traditional gel electrodes are not suitable for long term wearable applications due to the reduced performance over time due to moisture evaporation and faster contamination build-up. There are increasing levels of research activity focusing on integrating dry electrodes into textiles for wearable healthcare applications. However, most applications focus on diagnostics and monitoring such as ECG [14,15], EEG [16,17] and EMG [18,19]. Their application in therapeutics is limited mainly due to the issue of discomfort caused by the high impedance between the dry electrode and skin [20,21].

**Figure 1:**
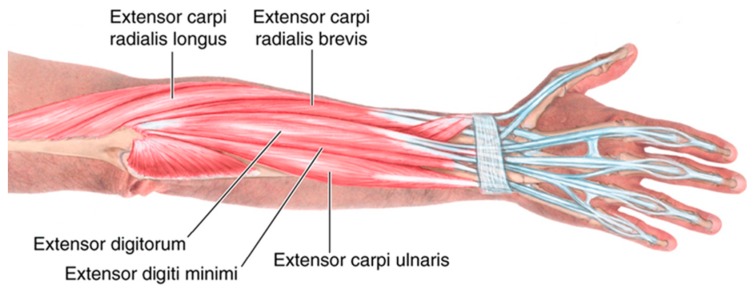
Muscle groups covered by the fabric electrode array.

This paper presents the development of a novel fabric electrode-based wearable training system for stroke rehabilitation through a co-design process with end users. Stroke is the leading cause of adult disability. Every year in the UK over 100,000 strokes occur, and worldwide this figure is 17 million [22]. Half of all stroke survivors have resulting arm/hand disability and need assistance with everyday tasks, which impacts on their quality of life. Intensive, repetitive, task-oriented training is beneficial for arm rehabilitation [23]. This can be delivered though FES, which is applied via electrodes on the skin to stimulate the underlying nerves. The stimulation contracts the muscle allowing the person to practice specific movements. Extensive motor learning and neurophysiology research, backed by substantial clinical evidence, underpins FES as an effective way of recovering movement, post-stroke [24,25,26]. However, there are several barriers to using FES, which limits uptake of this technology among stroke survivors. For example, current commercial FES devices use large gel electrodes that are very difficult to place in the correct location and which only stimulate a limited number of muscles. To overcome the limitation of using large electrodes, a gel electrode array with multiple elements which can be individually activated has been used in many studies [27,28,29,30]. This work has developed a 24-electrode array by printing an array of dry electrodes directly on a textile. The dry electrode developed in this work has a longer lifetime compared to the gel electrode because it can be washed and reused many times. The electrode array layout eliminates the need for accurate positioning as it covers a wide range of muscle groups (Figure 1) and the control algorithm used in this study can calculate the optimized combination of electrode elements to achieve targeted movements. The e-textile-based wearable training system developed in this work can facilitate intensive, repetitive and task-oriented training. While the initial tests were targeted on stroke rehabilitation, the technology can also be used for rehabilitation activities for subjects with other neurological disorders (e.g., Parkinson’s, Multiple Sclerosis, and Spinal Cord Injury).

## 2. Materials and Methods

### 2.1. Materials

The polyester/cotton (A1656, plain weaving, 165 μm thick) used in this study was purchased from Whaleys Bradford Ltd. (Bradford, UK). The functional pastes for screen printing listed in Table 1 were supplied by Smart Fabric Inks Ltd. (Southampton, UK). 

### 2.2. E-Sleeve Fabrication Process

#### 2.2.1. Fabric Electrode Array Fabrication

A DEK248 semi-automatic screen printer shown in

**Figure 2:**
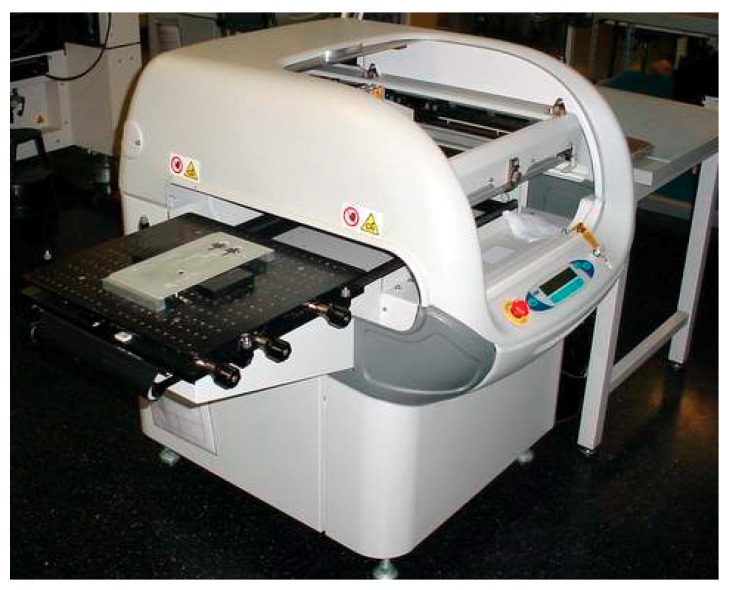
DEK 248 screen printer used in this work.

**Figure 3:**
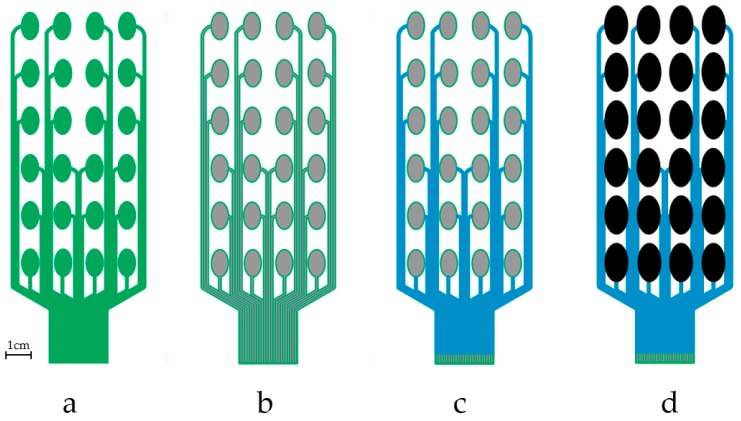
Top views of the FES processing after the printing of each layer, with sequence: interface layer (**a**); conductive silver layer (**b**); encapsulation layer (**c**); and electrode layer (**d**).

Figure 2 was used to print the fabric electrode array. The electrode array was fabricated by printing four functional layers as detailed below. The resulting patterns after printing each layer are shown in Figure 3.

Printing process used to produce the electrode array:

(1) Print interface layer to create a smooth and flexible surface on to which the silver paste can be printed. A 250 thread/inch stainless steel screen with 40 μm emulsion thickness supplied by MCI Precision Screens Ltd. (Melbourn, UK). was used. The interface layer was formed using two prints of Fabink IF-UV-1039 followed one print of Fabink IF-UV-1004. Using two types of interface provides waterproof properties and details have been published in our previous work [31]. UV curing was applied after each print by exposing the sample to a 400 W mercury (Hg) bulb in a UV cabinet supplied by UV Light Technology Ltd. (Birmingham, UK). The UV curing time was 60 s for Fabink IF-UV-1039 and 30 s for Fabink IF-UV1004. The total thickness of the interface is 170 μm.
(2) Print conductive layer to create the conductive tracks and conductive pads. A 120 thread/cm polyester screen with 10 μm emulsion thickness was used. The conductive layer was formed using one print of Fabink TC-C4007. The paste was cured in a box oven at 130 °C for 25 min. The thickness of the conductive silver layer is 8 μm.
(3) Print encapsulation to protect the conductive tracks and provide electrical insulation. A 250 thread/inch stainless steel screen with 30 μm emulsion thickness was used in the encapsulation layer printing. The encapsulation layer was formed using one print of Fabink IF-UV1004 followed by two print of Fabink IF-UV-1039 and cured as specified before. The thickness of the encapsulation layer is 110 μm.
(4) Print dry electrode to provide interface between the conductive pads and the skin. A thick stainless steel stencil screen was used in the electrode layer printing. The electrode paste was cured in a box oven at 80 °C for 30 min leaving a dry electrode layer with a thickness of 1 mm. In addition to the electrode layout shown in Figure 3d, three other types of electrodes layout were also fabricated and evaluated as discussed later in Section 3.1.

A more detailed description of the printing process is available in a previous publication [32].

#### 2.2.2. E-Sleeve Co-Design with End Users

The e-sleeve was made by sewing the fabric electrode array onto a piece of stretchable fabric which ensures a stable and tight, yet comfortable, contact between the electrodes and the skin. It is important that the final design of the e-sleeve meets the needs of the users. For example, for stroke survivors, the garment must be easy to don and doff using a single hand to enable independent use. Therefore, different garment designs including short and long cylindrical sleeves and a T-shirt configuration were assessed by the end user group consisting of a total of 10 stroke survivors and their carers. A ready-made cylindrical sleeve may simply be pulled on by the user. However, loop-and-hook fasteners and zip approaches may also be used to assemble the sleeve on the arm enabling a looser fitting sleeve to be put on and then tightened. The researcher met with each stroke survivor on two separate sessions. Stroke survivors were given prototype sleeves and example color samples and were asked to evaluate the different approaches to the sleeve design. This was achieved using a think-aloud method whereby stroke survivors commented on how easy it was to don and doff the sleeves and how comfortable they were. They were also asked closed questions regarding preferred sleeve design, color and washing technique.

There were 4 female and 6 male stroke survivors with an average age of 59 years (age ranged from 24–77 years). All the stroke survivors were in the chronic stages of stroke, and the time since stroke ranged from 2 years 3 months post-stroke to 11 years 4 months (average of 6 years 8 months). There was a range of stroke severity, with Fugl-Meyer upper limb scores ranging from 2 to 9 (average of 5.2). All the stroke survivors experienced problems with using their hand and upper limb, and needed assistance with everyday living tasks involving the upper limb (e.g., using a knife and fork).

### 2.3. Durability Test

**Figure 4:**
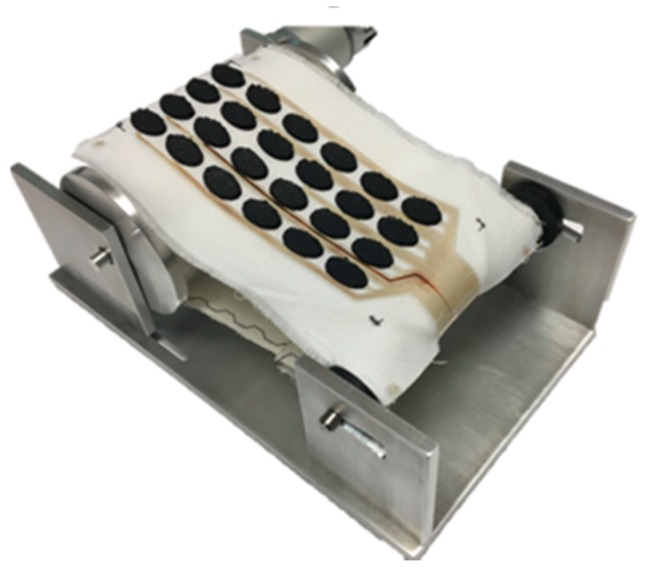
Bending test equipment.

Clothing experiences rigorous bending during everyday use, especially during donning and doffing, so it is important that any wearable electronics should survive these processes. To simulate this, bending durability was assessed by flexing the electrode array round a 10 mm radius for 1000 cycles using the bespoke equipment shown in Figure 4.

In addition to bending, an appropriate cleaning method for the e-sleeve is also required to remove any contamination and bacteria built up during wearing. Two cleaning methods (washing and wiping) were proposed to the end users and their feedback collected. For the washing method, a quick daily washing program (1400 rpm, 39 min) of a home use BEKO WME7247 washing machine was used and home laundry detergent was added for the washing test. For the wiping method, three types of wipe were evaluated including 5% SQ53 [33] wipes, Huggies Baby Wipes (99% water, fragrance free) and Wet Ones Wipes (original antibacterial). The bacterial level without and with cleaning was assessed using the following process:

(1) Electrodes were placed in a sterile tube containing tryptone soya broth and autoclaved glass beads.
(2) The tubes were vortex mixed for 2 min to remove any attached bacteria.
(3) Aliquots of 50 μL were spread over tryptone soya agar plates.
(4) Agar plates were incubated at 37 °C, overnight, before colonies were counted.

### 2.4. Muscle Stimulation Testing

**Figure 5:**
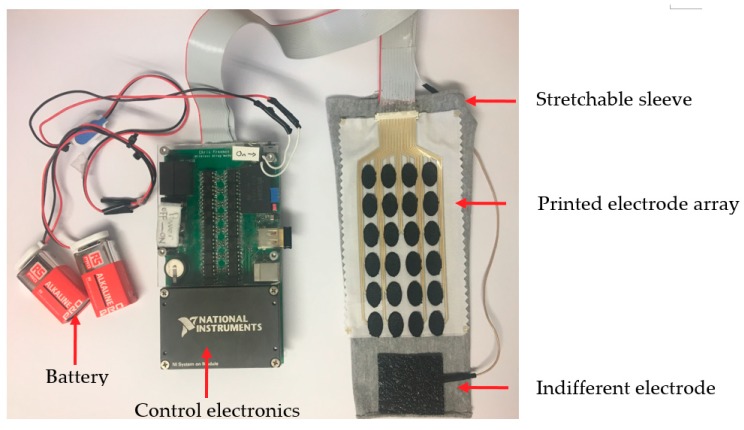
E-sleeve and electronics connection.

The FES training system includes an e-sleeve with electronics, training software and a movement sensor (Kinect v2.0 from Microsoft, Redmond, KS, USA). The electrode array on the e-sleeve was connected to the control electronics via a 24-way ribbon cable as shown in Figure 5. The control electronics provides a separate drive channel for each element in the 24-electrode array; each drive channel is controlled independently by the hardware. Each channel has an optically isolated constant-current output stage, with the current set to 9 mA. The pulse frequency of all channels was 40 Hz, and the pulse width of each channel is the controlled variable. The range of pulse width values is from 0 to 300 microseconds, with maximum value set at the start of the test. The stimulation voltage is constant and the same for all channels at 150 V DC.

**Figure 6:**
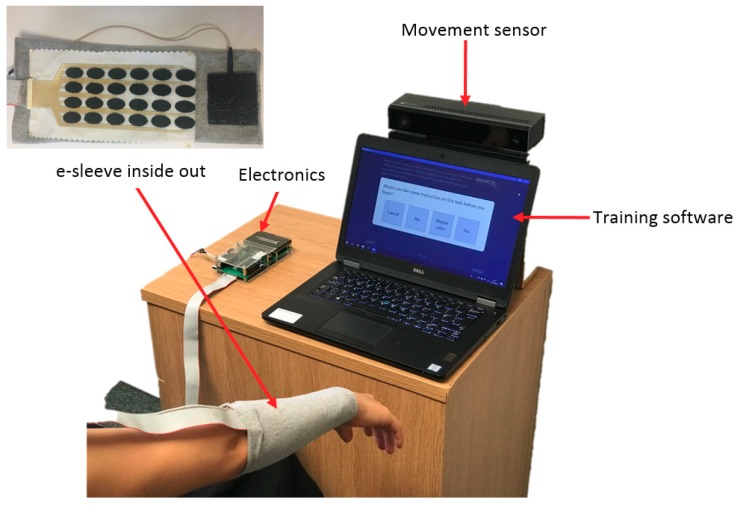
FES training system.

To perform the tests, the system was set up as shown in Figure 6. The following process was followed:

(1) At the beginning of the session, the pulse width applied to a central element in the array is slowly increased until a comfortable limit is found. This is set as the maximum pulse width that that can be applied to any channel for the individual user.
(2) The control algorithm then applies stimulation to each electrode in turn, using a triangular pulse width profile of 6 s duration. At the end of this profile the stimulation is removed and a further 1 s waiting period is provided to allow the hand to return to its initial position. During each profile, the angular movement produced by the hand and wrist is recorded using the movement sensor. This comprises 2 wrist angles and 10 finger angles. The slope of each joint angle is computed via least-squares fitting. When repeated over the 24 electrodes, this produces a 12 by 24 matrix which captures the static response to stimulation.
(3) For each test, the sensor then records the initial and desired final angular positions for the target gesture (e.g., hand opening, pointing).
(4) The control system then uses the desired movement (from (3)), together with the response matrix (from (2)) to compute a set of array elements and associated pulse width values which is best able to achieve the intended movement. For ease of computation, and to minimize the effect of nonlinearity, a set of only 3 elements is stipulated.

Further details of the optimization procedure can be found in previous publications [34,35]. 

## 3. Results and Discussion

### 3.1. Fabric Electrode Array and E-Sleeve Fabrication

**Figure 7:**
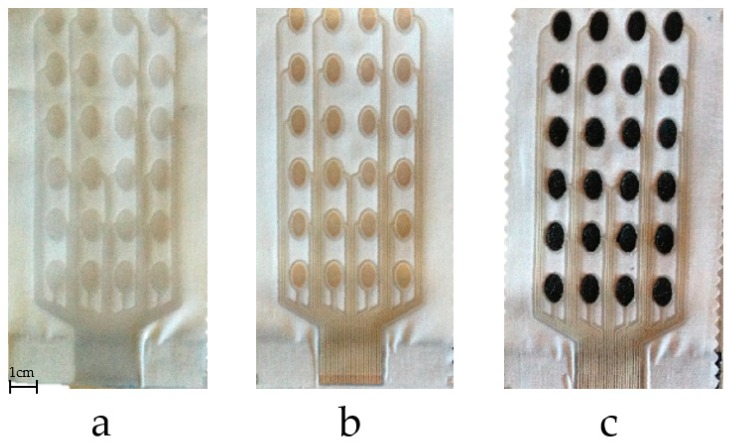
Fabric electrode array printing stages: interface layer (**a**); conductive and encapsulation layers (**b**); electrode layer (**c**).

**Figure 8:**
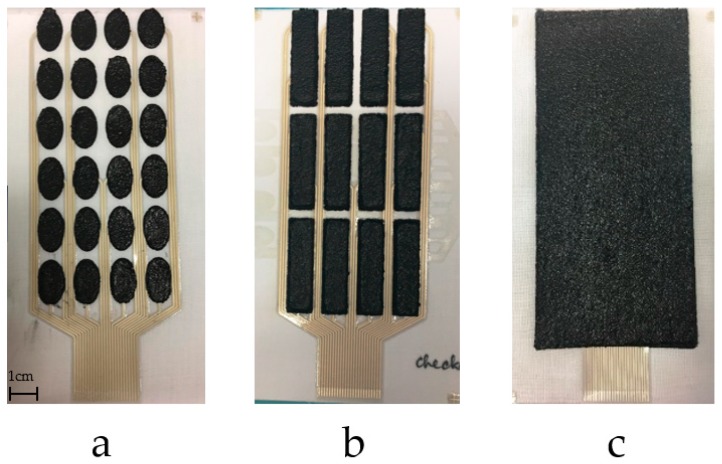
Alternative dry electrode layouts: 24 larger electrode elements (**a**); 12 electrode elements (**b**); 1 big electrode (**c**).

The printed samples, after printing each of the functional layers, are shown in Figure 7. The fabric electrode array maintains good flexibility and conformability. In addition to the electrode shown in Figure 7c, three different layouts have also been printed as shown in Figure 8. Different electrode array designs can be used to accommodate the needs of the different end users who may have different sensitivity to electrical stimulation and individual pain thresholds. While small electrode array elements maintain a high level of breathability of the textile, an array of larger electrodes provides improved levels of comfort when the stimulation intensity is increased.

The e-sleeve was co-designed with end users including stroke survivors and their carers. Three properties (color, length and assembly method) were evaluated and the feedback received is summarized below:

Color: Five colors as listed in Table 2 were assessed by nine end users for the outer of the e-sleeve. Gray and blue are the most popular followed by red then black. White was the least favorite color. However, all participants said the color did not really matter, and it is not a key element they would consider when they choose a device.

Length: The end users were asked that if the device need to cover both the lower arm and upper arm to stimulate all the necessary muscles, whether their preferred design was two short sleeves (one on the lower arm and one on the upper arm) or one long sleeve. 4 out of 10 preferred two shorter sleeves; 5 out of 10 preferred a long sleeve and 1 out of 10 had no preference. When they were also asked whether they would like the wearable e-sleeve to be integrated into their clothing or worn under their own clothing, they chose to wear it under clothing and not to integrate it into their everyday clothing.

Assembly methods: Three prototype e-sleeves with different assembly methods (pull-on, zip, and hook-and-loop) were assessed by seven end users. They all preferred the pull-on sleeve as it was easier to use, and they could use it by themselves without assistance from their carers. However, the zip version was found to be difficult to use and it was hard to align the parts of the hook-and-loop version.

**Figure 9:**
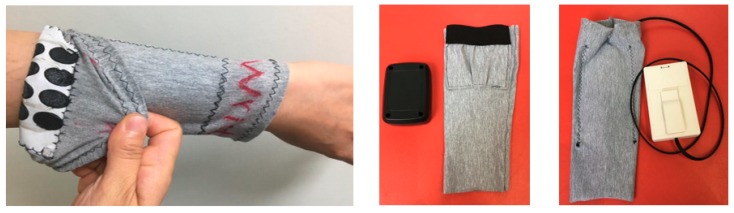
Pull-on sleeve with electrode array integrated (**left**); electronics mounting options: pocket (**center**); cable (**right**).

Based on the feedback from the end users, an optimized design shown in Figure 9 has been achieved. The methods for connecting the electrode array with the associated control electronics were also evaluated. Two options for connecting with the control electronics were proposed to the end users, as shown in Figure 9.

(1) The electronics was contained in a pocket that was included on the e-sleeve
(2) The electronics was connected via a lead and either clipped onto clothing, placed in a pocket or left on the table.

The two designs were both well received by the end users with no strong overall preference. 

### 3.2. Fabric Electrode Array Durability Tests

#### 3.2.1. Bending Test

The electrode array shows good durability to bending as there was no visible damage after 1000 bending cycles around a 10 mm radius. The resistance between the bottom conductive silver track and the carbon electrode also did not change.

#### 3.2.2. Cleaning Tests

**Figure 10:**
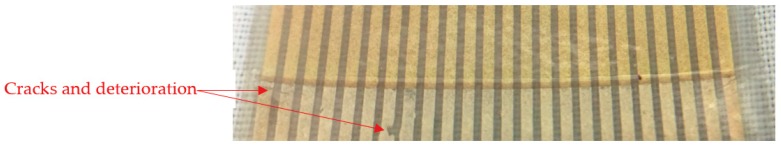
Silver tracks after washing for eight cycles.

Machine Washing: The fabric electrode array can survive up to eight washing cycles when it was put into a plastic wash ball which was used to minimize twisting of the fabric during the washing process. After eight wash cycles, the conductive tracks without the encapsulation layer (bottom part in Figure 10) started to crack and lost conductivity while the conductive tracks within the encapsulation layer (top part in Figure 10) remained undamaged.

**Figure 11:**
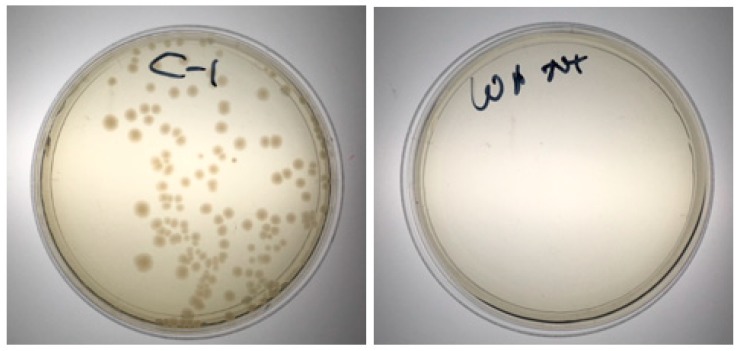
Bacteria colonies without (**left**) and with (**right**) cleaning by wiping.

Wiping: All three types of wipes tested (5% SQ53, Huggies Baby and Wet Ones) can remove bacteria effectively from the electrode array as shown in Figure 11. The wiping process did not cause any damage to the electrode array.

The two cleaning methods (machine washing and wiping) were proposed to nine end users. Five out of nine preferred to wash the array in a washing machine as they said it was easier and more hygienic; one of the nine preferred to hand wash the array because it would be easier; the remaining three out of the nine preferred to wipe the array because it would be quicker and easier. 

### 3.3. Muscle Stimulation Test

The e-sleeve was tested on eight stroke survivors with weak/disabled upper limbs. The maximum stimulation level (pulse width in microseconds) that was deemed comfortable by each participant is shown in Table 3. It is desirable to have the maximum stimulation level as high as possible as this affords more range for the control algorithm.

**Figure 12:**
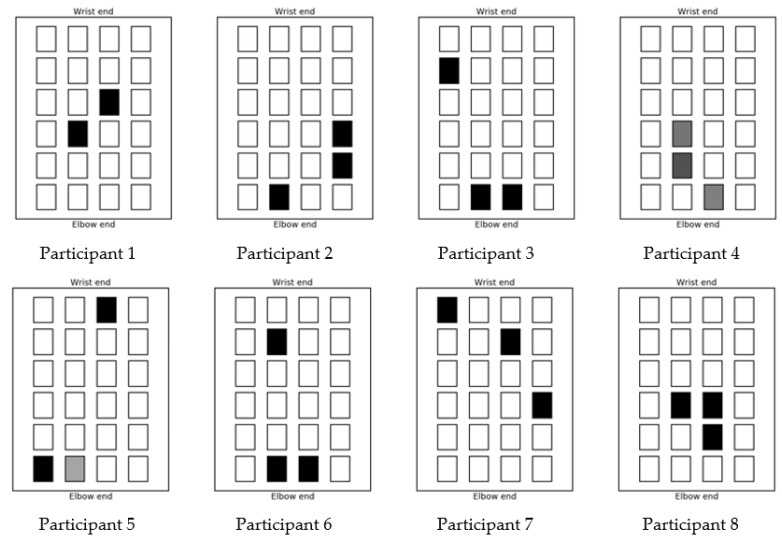
Stimulation patterns for hand opening gesture.

**Figure 13:**
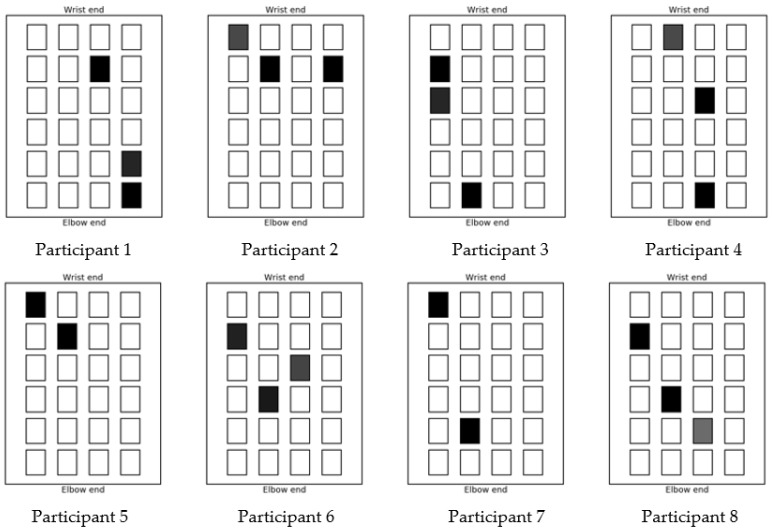
Stimulation patterns for pointing gesture.

Once the movements associated with each individual array element have been determined and recorded by the movement sensor the test system is ready to test the achievement of a specified hand gesture. In this work two gestures were attempted: the first is hand opening where the wrist is pulled back and the fingers are straight and splayed; the second gesture is pointing where the wrist is in a neutral position, the first finger is extended straight, and the remaining fingers are curled toward the palm of the hand. In each test, the starting position of the hand, without any stimulation applied, is recorded by the movement sensor. The desired finishing point and gesture is then recorded. To record this position, it may be necessary to place another person’s hand over the test subject’s hand to form the desired gesture if the stroke survivor cannot move their hand to the targeted gesture. Once the start and finish positions have been recorded, the control algorithm then calculates a combination of up to three array elements and their individual stimulation levels as a basis to recreate the desired finishing position. The selection of these array element and levels varies from person to person. The optimized selection of electrode array elements used to achieve the hand opening gesture for each participant are shown in Figure 12 and those used for the pointing gesture are shown in Figure 13. In both figures the depth of shading indicates the stimulation level, with black being the maximum comfortable level for that person. As can be seen from the figures, this confirms that the position of the optimized array elements varies from person to person; it also varies from gesture to gesture. The multi electrode array design and the control algorithm enables the system to choose optimized electrode elements for a specific user to achieve accurate movement with the minimum stimulation of the muscle area. This will reduce the muscle fatigue that is associated with FES when using larger electrodes. There was no discomfort reported from the stroke survivors.

## 4. Conclusions

A fabric electrode array with four functional layers has been manufactured using screen printing for use as part of an FES rehabilitation device. Four electrode layouts with different electrode shapes and sizes were achieved. The electrode array showed good bending durability being able to withstand the bending and flexing likely to occur during everyday use. The electrode array can be cleaned using machine washing and can also be easily cleaned by wiping the electrode surface with a range of cleaning wipes. Microbiology tests confirmed that bacterial contamination had been removed successfully for the surface wiping option. The electrode array must be integrated into an elastic fabric to form a clothing item for use, with the elastic fabric contracting to hold the electrode array in contact with the arm. The end user panel feedback ranked the pull-on sleeve as the preferred choice of design over the alternatives presented. Muscle stimulation to achieve targeted movements (e.g., pointing, hand opening) has been achieved by stimulating the optimized combination of electrode array elements, with the optimized combination automatically selected by the control algorithm. A user-friendly wearable training system including an e-sleeve with electronics, training software and movement sensor has been developed. The training system can facilitate rehabilitation exercises for stroke survivors to achieve targeted hand gestures and facilitate repeated movements as part of a rehabilitation program.

## Figures and Tables

**Table 1:** Pastes properties and curing conditions.

Pastes	Functionality	Curing Conditions
Fabink UV-IF-1004	Standard interface to create smooth surface on various fabrics	UV light, 30 s
Fabink UV-IF-1039	Waterproof interface and encapsulation suitable for various fabrics	UV light, 60 s
Fabink TC-C4007	Silver ink for printing flexible conductor layer on top of the interface layer	120–130 °C, 10–25 min
Fabink TC-E0002	Silicone rubber carbon paste for printing dry electrode on top of the conductive layer	80 °C, 30 min

**Table 2:** Color for the e-sleeve.

Color	Black	White	Gray	Red	Blue
					

**Table 3:** Maximum FES stimulation level for each participant.

Participant No.	1	2	3	4	5	6	7	8
Maximum comfort stimulation level	30	65	70	50	50	41	62	100
